# Electroencephalography in the Intensive Care Unit: Experience of the Department of Functional Neurosensory Explorations at Gonesse Hospital, France

**DOI:** 10.7759/cureus.95086

**Published:** 2025-10-21

**Authors:** Idrissa Doumbouya, Karinka Diawara, Elian Hapca, Djénabou Négué Barry, Mohamed Traoré, Gabriela Carelli, Oumar Sylla, Andreea Stanciulescu, Fodé Abass Cissé

**Affiliations:** 1 Department of Neurology, Ignace Deen Hospital, Gamal Abdel Nasser University, Conakry, GIN; 2 Department of Functional Neurosensory Explorations, Gonesse Hospital, Gonesse, FRA; 3 Department of Physiology, Functional Testing and Obesity Center, Louis-Mourier Hospital, Colombes, FRA; 4 Department of Clinical Physiology, Multidisciplinary Functional Testing and Sleep Center, Bichat-Claude Bernard Hospital, Paris, FRA

**Keywords:** eeg, epileptic seizure, gonesse, intensive care, neurophysiology

## Abstract

Introduction: The aim of this study was to evaluate the indications and outcomes of electroencephalography (EEG) in the intensive care unit (ICU), as well as to assess their prognostic values in adults.

Materials and methods: We conducted a two-year retrospective study from November 2022 to October 2024 in the Department of Functional Neurosensory Explorations at Gonesse Hospital, involving patients hospitalized in the ICU. Sociodemographic, clinical, electroencephalographic, and prognostic variables were collected. During data analysis, we investigated EEG patterns associated with poor prognosis and epileptic abnormalities.

Results: A total of 76 participants met our selection criteria, with a mean age of 61 ± 18.8 years and a predominance of men (sex ratio: 1.92). The main indications for EEG were neuroprognostic evaluation in 53 patients (68.5%), seizure assessment in 32 patients (40.8%), and unexplained neurological deterioration in four patients (5.3%). The mean duration of EEG recordings was 50 minutes; 17 patients (22.4%) experienced clinical seizures. Epileptic abnormalities were observed in 43 patients (56.6%), including electrographic seizures in 18 patients (23.7%), non-specific interictal activity in 10 patients (13.2%), non-convulsive status epilepticus (NCSE) in eight patients (10.5%), and lateralized periodic discharges (LPDs) in seven patients (9.2%). These findings accounted for 41.9%, 23.3%, 18.6%, and 16.3% of the epileptic abnormalities, respectively. Regarding non-epileptic abnormalities, 63 patients (82.9%) exhibited generalized slow waves, followed by focal slow waves in 27 patients (35.5%), generalized periodic discharges in 15 patients (19.7%), and burst-suppression patterns in 11 patients (14.5%). There was a statistically significant correlation between epileptic abnormalities and status epilepticus (p = 0.0003), ischemic stroke (p = 0.0317), and abnormal brain imaging (p = 0.0421). Poor prognostic factors included the absence of EEG reactivity (p = 0.0001), burst suppression (p = 0.0002), and the absence of cerebral activity (p = 0.0163).

Conclusion: EEG in the ICU setting not only provides valuable information regarding the epileptic origin of coma but also offers important prognostic insights.

## Introduction

Electroencephalography (EEG) visualizes brain activity by detecting differences in electrical potential between electrodes placed on the scalp over time, to aid in the diagnosis, management, and prognosis of brain pathologies [1]. EEG analysis, which traditionally relies on visual reading and clinically oriented interpretation by qualified physicians, plays an important role, from the quality control of the recording to advanced EEG interpretation [1]. In intensive care, EEG monitoring is an effective surveillance tool for detecting and characterizing epileptic seizures, assessing the severity of encephalopathy, evaluating sedation levels, and aiding prognosis [2]. It also plays a role in the detection and management of non-convulsive seizures (NCS) and non-convulsive status epilepticus (NCSE) [3], monitoring of neurosurgical patients, and classification of paroxysmal clinical events [4-6]. Recognizing epileptic seizures in the intensive care unit (ICU) is essential due to their impact on morbidity and mortality, as well as the risk of developing epilepsy following acute symptomatic events [7]. However, the use of EEG in the ICU presents challenges, particularly regarding the interpretation of results, which can be affected by patient sedation and artifacts inherent to the intensive care environment. While there is a growing body of literature describing EEG practices in academic hospital ICUs, little is known about EGG utilization patterns, diagnostic yield, and clinical impact in non-academic centers where resource limitations and staffing models may differ substantially [8]. Addressing this knowledge gap is essential to guide the rational implementation of EEG monitoring and improve timely seizure detection and the accessibility of neurodiagnostic care. Therefore, the aim of this study was to evaluate the indications and outcomes of EEG in the ICU at a general hospital in the suburb of Paris, as well as to assess their prognostic value in adults.

## Materials and methods

We conducted a two-year retrospective study from November 2022 to October 2024 in the Department of Functional Neurosensory Explorations at Gonesse Hospital. The department operates from Monday to Friday during standard working hours (50 hours/week) and is equipped to perform EEG recordings at night when necessary. EEG recordings were performed using a BRAIN QUICK system (Natus Medical Incorporated, Middleton, WI) equipped with analysis and reporting software and an SD PLUS amplifier, both of which were used throughout this study. Recordings were carried out by qualified nurses in accordance with the international 10-20 electrode placement system and in compliance with French national EEG guidelines [9]. EEGs were examined and pre-analyzed by a trained neurologist and then reinterpreted and validated by a neurophysiologist to ensure consistent and rigorous interpretation.

We included all EEGs conducted on comatose patients admitted to the ICU. Patients under 18 years of age, as well as those with missing critical data, particularly regarding EEGs or essential clinical variables, were excluded. For each patient, the following data were collected.

Sociodemographic data included patient age and sex. Clinical data encompassed the reason for ICU admission, associated comorbidities, and the indication for EEG. Patients were classified based on whether or not they had experienced seizures, and the type of seizure was recorded.

EEG recordings were performed intermittently on all patients by the same technical team. EEG findings were categorized as either epileptic or non-epileptic. Seizure diagnosis during EEG was based on the criteria established by Chong and Hirsch [10]. Rhythmic and periodic patterns, including lateralized periodic discharges (LPDs) and generalized periodic discharges (GPDs), were defined according to the American Clinical Neurophysiology Society (ACNS) Standardized Critical Care EEG Terminology: 2021 version [11]. Non-convulsive status epilepticus (NCSE) was defined using the Salzburg criteria [12]. Other EEG patterns were classified as normal, slowed, or inactive. EEG duration was calculated by summing the total recording time for each patient. The use of anti-seizure medications was also documented.

Brain imaging findings were classified as either normal or abnormal, and patient outcomes, including mortality, were recorded.

The data were extracted from the electronic medical records of comatose patients admitted to the ICU, including clinical records and EEG results obtained during their hospitalization. Cases of missing data were systematically noted. To ensure the quality of the data collected, regular checks were performed on a random sample of medical records, and audits were conducted to detect any inconsistencies or missing important data.

All data were anonymized to protect confidentiality in accordance with the principles of the Declaration of Helsinki. The study has been approved by the local ethics committee.

Data analysis was performed using Epi Info™ version 7.1, a database and statistical software developed by the Centers for Disease Control and Prevention (CDC, Atlanta, GA). Quantitative variables were expressed as means, and qualitative variables were presented as frequencies and numbers. Comparisons between qualitative variables were made using the chi-square test (a nonparametric test), while comparisons between quantitative variables were performed using Student’s t-test (a parametric test). A p-value of less than 0.05 was considered statistically significant.

## Results

Out of a total of 3,900 patients who underwent EEG in the department, 77 patients were from the intensive care unit. One patient was under 18 years of age and was therefore excluded, resulting in a final sample of 76 participants, representing 1.9% of all EEGs performed.

The overall mean age was 61 ± 18.8 years, with a median of 63 years and extremes of 18 and 100 years. The mean age was 62 for women and 60 for men. There was a predominance of men, with a sex ratio of 1.92 (50 men versus 26 women). There was no statistically significant difference between age and sex (p = 0.7382).

The main indications for EEG were neuroprognostic assessment in 53 patients (68.5%), search for seizures in 32 patients (40.8%), and unexplained neurological deterioration in four patients (5.3%).

Reasons for admission to the intensive care unit were cardiorespiratory arrest in 29 patients (38.2%), ischemic stroke in 13 patients (17.1%), status epilepticus in 11 patients (14.5%), septic shock in 10 patients (13.2%), confusion in nine patients (11.8%), and cerebral hemorrhage in four patients (5.3%). Hypertension was the most common comorbidity, found in 33 patients (43.4%); other comorbidities were dominated by heart disease in 22 patients (28.9%), diabetes in 19 patients (25%), dyslipidemia in 13 patients (17.1%), stroke in eight patients (10.5%), and psychiatric disorders in seven patients (9.2%) (Table 1).

**Table 1:** Patients characteristics EEG, electroencephalography; ICU, intensive care unit

Variables	Number (n = 76)	Proportion (%)	
Reasons for performing an EEG			
Search for epileptic seizures	32		40.8
Neuroprognosis assessment	53		68.5
Unexplained neurological deterioration	4		5.3
Reasons for admission to the ICU			
Cardiorespiratory arrest	29		38.2
Ischemic stroke	13		17.1
Status epilepticus	11		14.5
Septic shock	10		13.2
Confusion	9		11.8
Cerebral hemorrhage	4		5.3
Comorbidities			
Hypertension	33		43.4
Heart disease	22		28.9
Diabetes	19		25
Dyslipidemia	13		17.1
Stroke	8		10.5
Psychiatric disorders	7		9.2
None	6		7.9
Epilepsia	6		7.9
Neoplasia	5		6.6
Alcoholism	4		5.3
Renal insufficiency	4		5.3
Smoking	3		3.9
HIV	3		3.9
Others	3		3.9

Clinically, 17 patients (22.4%) experienced seizures. Among them, six patients (7.9%) had generalized seizures, five patients (6.6%) had focal seizures with secondary bilateralization, three patients (3.9%) had focal seizures without bilateralization, and three patients (3.9%) had seizures of unknown onset.

In total, 34 patients (44.7%) had received at least one anti-seizure medication prior to EEG recording.

The mean EEG duration was 50 minutes, with a range from 20 to 1,420 minutes. In terms of electroencephalographic findings (Table 2), epileptic abnormalities were observed in 43 patients (56.6%). These included electrographic seizures in 18 patients (23.7%), non-specific interictal activity in 10 patients (13.2%), NCSE in eight patients (10.5%), and LPDs in seven patients (9.2%). These findings accounted for 41.9%, 23.3%, 18.6%, and 16.3% of the epileptic abnormalities, respectively.

**Table 2:** Patient EEG results NCSE, non-convulsive status epilepticus; LPDs, lateralized periodic discharges; EEG, electroencephalography

Variables	Number	Proportion (%)
Generalized slow waves	63	82.9
Focal slow waves	27	35.5
Generalized periodic activities	15	19.7
Burst suppression	11	14.5
Epileptic activity	43	56.6
Electrographic seizures	18	23.7
Non-specific interictal activity	10	13.2
NCSE	8	10.5
LPDs	7	9.2
Absence of cerebral activity	7	9.2
Normal	2	2.6
Coma alpha pattern	1	1.3

**Figure 1:**
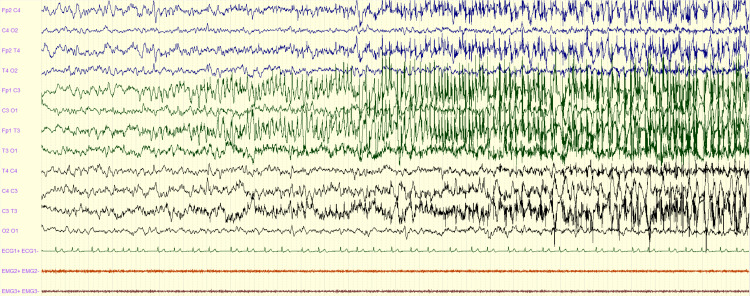
Left focal seizure with bilateralization: EEG showing left frontal rhythmic spikes with secondary bilateralization EEG: electroencephalography

**Figure 2:**
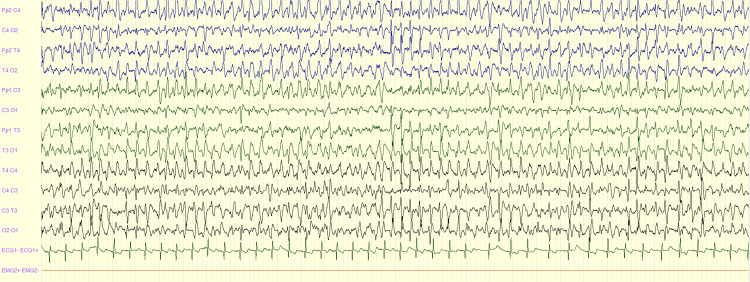
NCSE: generalized rhythmic epileptiform discharges after non-status epilepticus NCSE: non-convulsive status epilepticus

**Figure 3:**
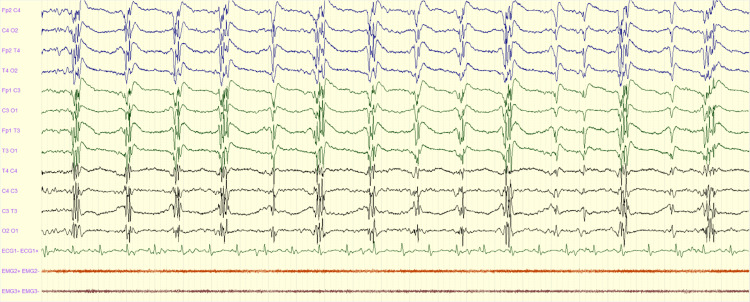
Generalized periodic activity: EEG showing periodic polyspikes in a patient with post-anoxic coma following cardiac arrest EEG: electroencephalography

**Figure 4:**
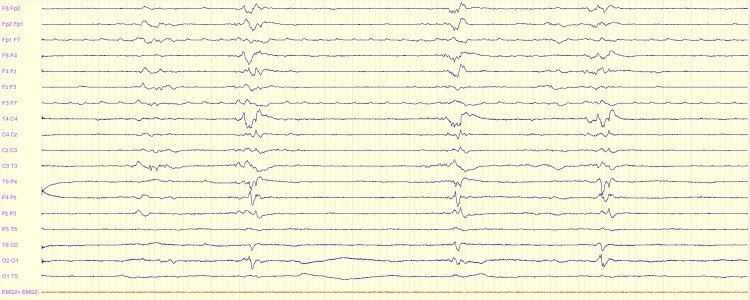
EEG showing a burst-suppression pattern in a patient after post-anoxic encephalopathy EEG: electroencephalography

Among non-epileptic abnormalities, the most frequent patterns were generalized slow waves in 63 patients (82.9%), focal slow waves in 27 patients (35.5%), generalized periodic discharges in 15 patients (19.7%), and burst-suppression patterns in 11 patients (14.5%) (Figures 1-4).

Fifty-three patients (69.7%) had abnormal brain imaging findings, with anoxic-ischemic lesions accounting for 19 patients (25%). Other brain abnormalities were infarctions in 13 patients (17.1%), cerebral edema in 12 patients (15.8%), and intracranial hemorrhage in nine patients (11.8%).

A statistically significant correlation was found between epileptic EEG abnormalities and the status epilepticus (p = 0.0003), ischemic stroke (p = 0.0317), and abnormal brain imaging (p = 0.0421) (Table 3).

**Table 3:** Epileptic abnormalities and patient characteristics

Variables	Epileptic abnormalities (%)		Total	χ^2^	P-value
	Yes	No			
Cardiorespiratory arrest	11 (37.9)	18 (62.1)	29	0.7725	0.3794
Status epilepticus	11 (100)	0	11	12.6344	0.0003
Ischemic stroke	10 (76.9)	3 (23.1)	13	4.6100	0.0317
Septic shock	2 (20)	8 (80)	10	0.8448	0.3580
Confusion	6 (66.7)	3 (33.3)	9	0.9318	0.3343
Abnormal brain imaging	30 (56.6)	23 (43.5)	46	4.1289	0.0421

A total of 28 patients (36.8%) died during the study period. Several factors were significantly associated with poor prognosis, including the use of more than two anti-seizure medications (p = 0.0156), the absence of EEG reactivity (p = 0.0001), burst-suppression pattern on EEG (p = 0.0002), and the absence of cerebral activity (p = 0.0163) (Table 4).

**Table 4:** Poor prognostic factors EEG: electroencephalography

Variables	Death, n (%)		Total	χ^2^	P-value
	Yes	No			
Sedation	13 (48.2)	14 (51.8)	27	0.8159	0.3663
Number of anti-seizure medications					
1-2	9 (37.5)	15 (62.5)	24	5.8442	0.01562
3-5	9 (90)	1 (10)	10		
Abnormal brain imaging	23 (47.9)	25 (52.1)	48	4.6361	0.0175
Nonreactive EEG	25 (58.1)	18 (41.9)	43	17.2538	0.0001
Epileptic abnormalities	14 (40)	21 (60)	27	0.0834	0.7727
Burst suppression	9 (90.9)	1 (9.1)	11	13.5553	0.0002
Absence of cerebral activity	6 (85.7)	1 (14.3)	7	5.7700	0.0163
Generalized slow waves	21 (53.9)	42 (46.1)	63	1.1669	0.2800
Focal slow waves	12 (44.4)	15 (55.6)	27	0.5951	0.4404
Generalized periodic activities	5 (30.8)	10 (69.2)	15	0.0334	0.7571

## Discussion

The use of EEG in the ICU represents a significant advancement in the management of comatose or neurologically unstable patients, allowing for the early detection of seizures that may go clinically unnoticed. We conducted a 24-month retrospective study of EEG monitoring in the ICU setting. However, this study has several limitations. These include its retrospective design, a relatively small sample size that may limit statistical power, and the generalizability of the findings to other institutions. Additionally, the lack of systematic long-term EEG monitoring may have led to the underdetection of delayed or intermittent abnormalities.

There is a substantial body of literature on EEG monitoring in the ICU. However, in non-academic hospitals such as ours, where resources may be more limited and expertise in neurophysiology less readily available, the implementation of EEG monitoring in the ICU remains a challenge.

The prevalence of clinical seizures (22.4%) found in our study is consistent with the literature, where the reported prevalence of seizures in ICU patients varies widely, ranging from 7% to 47% depending on the study population and center [13,14]. Reported rates of non-convulsive status epilepticus (NCSE) also vary considerably, from 1% to 32% [13]. Claassen et al. demonstrated that nearly 90% of seizures in comatose ICU patients are non-convulsive and therefore would not be detected without EEG monitoring, highlighting the essential role of EEG in this setting [14]. The seizure frequency observed in our study may be partly explained by the relatively short duration of EEG recordings, which averaged 50 minutes, and by the use of anti-seizure medications. In practice, treatment decisions regarding anti-seizure medications are often made in real time, based on clinical suspicion, and do not necessarily wait for EEG confirmation. This practice could account for the relatively high proportion of patients (44.7%) who received at least one anti-seizure medication prior to EEG.

Epileptic abnormalities were identified in 43 patients (56.6%). Among them, electrographic seizures were observed in 18 patients (41.9%), followed by non-specific interictal activity in 10 patients (23.3%), LPDs in seven patients (16.3%), and non-convulsive status epilepticus (NCSE) in eight patients (18.6%). Claassen et al. reported an 18% frequency of electrographic seizures, and periodic epileptiform discharges were recorded in 19% of patients (LPDs, 13%; GPDs, 6%) [15]. In many patients with convulsive status epilepticus (CSE), electrographic seizures may persist even after convulsive activity has ceased [16]. In the Veterans Affairs Cooperative Study, Treiman et al. reported that patients who developed NCSE following convulsive status epilepticus (CSE) had more than twice the mortality compared to those whose seizures had completely ceased [17]. Similarly, in a prospective study, DeLorenzo et al. found that 48% of patients monitored by EEG for 24 hours after the resolution of CSE experienced non-convulsive seizures, and 14% developed NCSE [18].

Another common indication for EEG monitoring was neuroprognostic evaluation. Standard EEG, continuous EEG, and evoked potentials (particularly somatosensory evoked potentials) are frequently used to assess prognosis in cases of suspected irreversible neuronal damage [5]. In our study, non-epileptic EEG abnormalities were predominantly characterized by generalized slow waves, followed by focal slow waves, generalized periodic discharges, and burst-suppression patterns. Young and Mantia described a series of EEG changes correlated with various markers of multi-organ failure [19]. The initial abnormalities involved slowing in the theta range (>4 but <8 Hz), followed by delta activity (≤4 Hz), which progressed from intermittent and rhythmic to continuous and arrhythmic patterns. These were followed by the appearance of triphasic waves and, ultimately, a burst-suppression pattern. As in our study, their findings demonstrated a strong and nearly linear correlation between EEG deterioration and increased mortality.

Poor prognostic EEG factors were the lack of reactivity (p = 0.0001), burst-suppression patterns (p = 0.0002), anti-seizure medications exceeding two (p = 0.0156), and the lack of brain activity (p = 0.0163). In another study, Young et al. reported that in anoxic-ischemic encephalopathy, certain EEG patterns are strongly predictive of poor outcomes, with patients failing to regain consciousness and either remaining in a vegetative state or dying without recovery [20]. These patterns include the absence of cerebral activity, burst suppression with generalized epileptiform activity in bursts, and periodic generalized epileptiform discharges on a background of cerebral inactivity [20]. The severe outcomes correlated with burst suppression and complete electrocerebral inactivity in our study emphasize the necessity of EEG evaluation for accurate prognostication in such patients. In addition, other authors have reported that the absence of EEG reactivity to stimulation (defined as a lack of change in amplitude or frequency following a stimulus) also holds significant prognostic value and is associated with increased mortality or severe disability in most cases [21,22].

## Conclusions

EEG monitoring in the intensive care setting provides valuable insights into both the epileptic nature of coma and patient prognosis. In our study, a significant number of electrographic seizures went undetected clinically, which strongly supports the need for implementing continuous EEG monitoring even in peripheral or non-academic centers. Such monitoring can improve seizure detection, guide treatment decisions, and potentially improve patient outcomes. However, our study had limitations, including its retrospective design and relatively short EEG recording durations. Future studies should incorporate additional factors such as the degree of sedation, anti-seizure medication dosages, and extending continuous EEG monitoring beyond 24 hours. These investigations would offer a more comprehensive understanding of continuous EEG utility in the ICU setting, tailored to the specific constraints and resources of centers similar to ours.
